# Plasma anti-PRTN3 IgG and IgM autoantibodies: novel biomarkers for early detection of lung adenocarcinoma

**DOI:** 10.3389/fimmu.2025.1534078

**Published:** 2025-02-14

**Authors:** Yutong Li, Linhong Wang, Fengqi Chen, Rulan Liao, Jing Li, Xiaobin Cao, Songyun Ouyang, Liping Dai, Renle Du

**Affiliations:** ^1^ Henan Institute of Medical and Pharmaceutical Sciences, Zhengzhou University, Zhengzhou, Henan, China; ^2^ Beijing Genomics Institution (BGI) College, Zhengzhou University, Zhengzhou, Henan, China; ^3^ Henan Key Medical Laboratory of Tumor Molecular Biomarkers, Zhengzhou University, Zhengzhou, Henan, China; ^4^ Department of Respiratory and Critical Care Medicine, the First Affiliated Hospital of Zhengzhou University, Zhengzhou, China; ^5^ Henan Key Laboratory of Tumor Epidemiology & State Key Laboratory of Esophageal Cancer Prevention, Zhengzhou University, Zhengzhou, Henan, China; ^6^ Henan Key Laboratory for Pharmacology of Liver Diseases, Zhengzhou University, Zhengzhou, Henan, China

**Keywords:** autoantibody, biomarker, lung adenocarcinoma, PRTN3, early diagnosis

## Abstract

**Background:**

Proteinase 3 (PRTN3) has been recognized as a crucial target for anti-neutrophil cytoplasmic autoantibody. However, the relationship between anti-PRTN3 autoantibody and cancer remains largely unexplored.

**Methods:**

Immunohistochemistry was used to detect the level of PRTN3 in lung adenocarcinoma (LUAD) tissue array. Enzyme-linked immunosorbent assay was conducted to measure anti-PRTN3 IgG and IgM autoantibodies in plasma from patients with early- and advanced-stage LUAD, benign pulmonary nodules (BPN) and normal control (NC). Western blotting and immunofluorescence staining were performed to confirm the presence of plasma immune response to PRTN3.

**Results:**

PRTN3 protein was highly expressed in LUAD tissues. Elevated plasma levels of anti-PRTN3 IgG and IgM autoantibodies were also detected in LUAD, especially in early LUAD. The AUC of anti-PRTN3 IgG autoantibodies in the diagnosis of early LUAD from NC was 0.782, and from BPN was 0.761. When CEA and anti-PRTN3 autoantibodies were combined, the AUC for the diagnosis of early LUAD was significantly higher than that of CEA alone. The presence of a plasma immune response to PRTN3 in LUAD was also confirmed.

**Conclusion:**

Anti-PRTN3 IgG and IgM autoantibodies maybe early biomarkers to differentiate LUAD from NC and BPN.

## 1 Introduction

In recent decades, lung cancer (LC) is the leading cause of cancer death globally (1, 2). Specifically, lung adenocarcinoma (LUAD) is the most common subtype of LC (3). The early-stage of LC holds the greatest potential for therapeutic intervention. However, it does not exhibit any signs or symptoms, resulting in delayed diagnosis in the advanced-stage LC (4). It has been reported that only 16% of LC patients are diagnosed at an early stage (5). Low-dose CT (LDCT) is currently the most effective method for early diagnosis of LC. However, LDCT still struggles to accurately determine whether screened pulmonary nodules are benign and malignant (6). This often leads to unnecessary follow-up CT scans, additional tests, biopsies, and even surgery, causing an unnecessary mental and financial burden on patients. Traditional methods for LUAD detection involve the use of tumor markers (TMs). According to reports, CEA levels are abnormally elevated in 40% of LUAD patients, making CEA a specific marker for LUAD diagnosis (7). However, it lacks the enough sensitivity and specificity to detection and diagnose LUAD in clinical settings or serve as an early biomarker for LUAD patients (8). Therefore, the development of a non-invasive LUAD biomarker that can differentiate between benign and malignant pulmonary nodules is essential for improving prognosis and reducing the risk of overdiagnosis.

Since the early stage of cancer activates the body’s immune surveillance function (9), the immune system is capable of triggering specific host immune responses against tumor-associated antigens (TAAs). This results in the presence of corresponding autoantibodies against tumor-associated antigens (TAAbs), which can be detected in the plasma (10). TAAbs are stable and persist at high levels in the plasma of cancer patients for extended periods, especially in comparison to their absence in normal individuals and non-cancerous conditions (9). This suggests that TAAbs may serve as immunodiagnostic markers for the early diagnosis of cancer (11, 12). TAAbs generally include adaptive IgG or IgM, as well as other isotypes (13). However, recent reports on TAAbs biomarkers have primarily focused on studying IgG isotype autoantibodies. It should be noted that IgM not only forms part of the body’s first line of defense but also plays a role in recognizing precancerous and cancer cells (14–16). Therefore, it is essential to explore both IgG and IgM autoantibodies as cancer diagnostic biomarkers to ensure broad clinical application.

Inflammation is considered one of the main factors contributing to the production of TAAbs in cancer patients (17, 18). PRTN3, also known as myeloblastic protein or cytoplasmic pattern of antineutrophil cytoplasmic autoantibodies (c-ANCA) antigen, is a hematopoietic serine protease stored in the cytoplasmic azurophilic granules of neutrophils. It is involved in various inflammatory diseases (19). In granulomatosis with polyangiitis, anti-PRTN3 autoantibodies may induce vasculitis by recognizing membrane-bound PRTN3 (20). Moreover, PRTN3 is used as a biomarker for ulcerative colitis and primary sclerosing cholangitis in children (21). There have been documented associations between PRTN3 protein and cancer progression (22–25). However, the relationship between anti-PRTN3 autoantibodies and LUAD remains largely unknown.

In this study, we investigated the levels of anti-PRTN3 autoantibodies (IgG and IgM) in a large plasma sample consisting of individuals with LUAD, normal control (NC) and benign pulmonary nodules (BPN). Our aim was to determine the potential significance of anti-PRTN3 autoantibodies as novel early biomarkers for distinguishing LUAD from BPN and NC.

## 2 Materials and methods

### 2.1 Immunohistochemical assay

The human LUAD tissue array was obtained from Bioaitech Co. LTD (No. R076Lu01, Xian, China). Anti-PRTN3 antibody (Abcam, ab103632, Cambridge, UK) was provided as the primary antibody. All IHC analysis results were obtained from two independent pathologists. The degree of immunostaining was scored based on the intensity of positivity and the percentage of positive cells. The IHC scores were calculated as the positive intensity score multiplied by the percentage of positive cells, ranging from score 0 to 12. The intensity of positivity was graded according to the following criteria: negative score was 0; weak positive score was 1; moderate positive score was 2; strong positive score was 3. The percentage of staining positive cells was scored as follows: score 1 (1 ~ 25% positive), score 2 (26 ~ 50% positive), score 3 (51 ~ 75% positive), score 4 (76 ~ 100% positive).

### 2.2 Plasma from patients and controls

Plasma samples of two independent sets (training set and validation set) were used and detailed characteristics were shown in **Table 1**. The training set consisted of 95 LUAD patients and 98 NC matched by sex and age. In addition, 275 LUAD patients, 223 matched BPN patients, 82 matched lung squamous cell carcinoma (LUSC) patients and 275 matched NC were included in the validation set. All samples were collected from November 2019 to May 2022 from the First Affiliated Hospital of Zhengzhou University. Plasma samples from all patients were taken at the time of the patients’ first diagnosis and the patients were free of any other cancer, antineoplastic therapy, and autoimmune disease. All NC were healthy physical examination individuals with no history of cancer, pulmonary disease, or autoimmune disease. Plasma was extracted and stored according to standard protocols (26). The study was approved by the Medical Ethics Committee of Zhengzhou University, and all patients and NC signed an informed consent form before participating in the study.

**Table 1:** The characteristics of plasma subjects.

Variables	Training set			Validation set				
	NC (n=98)	LUAD (n=95)	P	NC (n=275)	BPN (n=223)	LUAD (n=275)	LUSC (n=82)	P
Age (year)			>0.05					>0.05
≥55	62 (63.3)	54 (56.8)		158 (57.5)	123 (55.2)	157 (57.1)	48 (58.5)	
<55	36 (36.7)	41 (43.2)		117 (42.5)	100 (44.8)	118 (42.9)	34 (41.5)	
Mean ± SD	55 ± 8	59 ± 11		56 ± 11	55 ± 12	56 ± 11	56 ± 11	
Gender, n (%)			>0.05					>0.05
Male	57 (58.2)	55 (57.8)		160 (58.2)	139 (62.3)	160 (58.2)	46 (56.1)	
Female	41 (41.8)	50 (42.1)		115 (41.8)	84 (37.6)	115 (41.8)	36 (43.9)	
Diameter, n (%)								
≤30mm		31 (32.6)			127 (56.9)	193 (70.2)	14 (17.1)	
>30mm		57 (60.0)			44 (19.7)	56 (20.4)	8 (0.09)	
Unknown		7 (7.4)			52 (23.3)	26 (9.4)	60 (73.2)	
Lymph node metastasis, n (%)								
Yes		45 (47.4)				63 (22.7)	43 (52.4)	
No		47 (49.5)				191 (69.4)	33 (40.2)	
Unknown		3 (3.2)				21 (8.0)	6 (0.0.7)	
Distant metastasis, n (%)								
Yes		29 (30.5)				28 (10.2)	10 (12.2)	
No		58 (61.1)				218 (79.3)	57 (69.5)	
Unknown		8 (8.4)				29 (10.5)	15 (18.3)	
Clinical stage, n (%)								
I		36 (37.8)				168 (61.0)	18 (21.9)	
II		15 (15.7)				15 (5.4)	9 (11.0)	
III		13 (13.6)				34 (12.3)	25 (30.5)	
IV		28 (29.4)				26 (9.4)	8 (0.10)	
Unknown		3 (3.1)				32 (11.6)	22 (26.8)	

LUAD, lung adenocarcinoma; BPN, benign pulmonary nodule; NC, normal control.

### 2.3 Enzyme−linked immunosorbent assay

The recombinant PRTN3 protein was obtained from Cloud-Clone Corp (No. RPB434Hu01, Wuhan, China). In brief, recombinant human protein PRTN3 was coated to 96-well microliter plates as antigens. Plates were then blocked with 2%BSA. The plasma samples were used as the primary antibody at the dilution of 1:100. The secondary antibodies were goat anti-human IgG and goat anti-human IgM conjugated horseradish peroxidase (HRP) (Wuhan Aoko Biotechnology Co. LTD), which was diluted at 1:5000. Blank control and quality control were set on each plate to ensure the assay quality. The blank control was defined as no antibody was added and all other steps were the same. The quality control plasma was an equal mixture of 100 NC plasma samples. The diagnosis value was expressed as specific binding index (SBI), which was calculated by the formula: SBI= (OD value of test plasma sample - OD value of blank control sample)/(OD value of quality control sample - OD value of blank control sample).

### 2.4 Western blotting

The plasma samples of LUAD patients with positive ELISA results, BPN patients and NC with negative ELISA results were randomly selected for western blotting to further verify the ELISA results. To ensure uniform testing conditions for all plasma samples, the recombinant protein PRTN3 (21kD) underwent denaturation at 99°C. It was then separated using SDS-PAGE and transferred onto a PVDF membrane. The PVDF membrane was subsequently cut into 16 strips and incubated with the corresponding 1:100 dilution of plasma or a 1:500 dilution of anti-PRTN3 antibody. As a positive control, rabbit monoclonal anti-PRTN3 antibody was utilized. Following this, the plasma samples were incubated with goat anti-human IgG conjugated to horseradish peroxidase (HRP) at a dilution of 1:2500. For the secondary antibody of the positive control, goat anti-rabbit IgG was used. By denaturing and transferring the recombinant protein, followed by cutting the PVDF membrane into strips and using specific dilutions for the plasma samples and antibodies, the immune response to PRTN3 could be accurately detected.

### 2.5 Immunofluorescence staining

LUAD patients with the strongest immunoreactivity to PRTN3 recombinant protein in western blotting and NC plasma with negative expression were selected for IF staining. LUAD and NC plasma were preabsorbed with recombinant PRTN3 protein, and the IF signal before and after PRTN3 absorption was detected by IF. A549 cells and H1299 cells in logarithmic growth phase were allowed to grow on a cover slip. The cells were fixed with 4% paraformaldehyde, permeabilized with 0.2% TritonX-100, blocked with 1% BSA, and then incubated with the plasma (diluted at 1:100) overnight. Rabbit monoclonal anti-PRTN3 antibody was regarded as a positive control. FITC-labeled goat anti-human IgG was used as the corresponding secondary antibody for plasma, and goat anti-rabbit IgG was used as the secondary antibody for the positive control. Finally, the slides were sealed with anti-fluorescence quench sealant containing DAPI. All images were acquired under the same conditions.

### 2.6 Statistical analysis

SPSS Statistics 26.0 and GraphPad Prism 9.5 software were used for data analysis and visualization. IHC scores in IHC were analyzed by independent t-test. In addition, Mann-Whitney U test was used to analyze the difference of autoantibodies levels between different populations. χ2 test was used to evaluate the difference of autoantibodies positive rates. Logistic regression was used to establish a combined diagnostic model of autoantibodies and TMs. The area under the curve (AUC) and 95% confidence interval (CI) of receiver operating characteristic (ROC) were used to evaluate the diagnostic performance of biomarkers and models. The SBI value at the maximum Youden’s index was used as the cut-off value to determine the sensitivity and specificity of diagnosis. In all tests, P < 0.05 (two-tailed) was considered statistically significant.

## 3 Results

### 3.1 PRTN3 protein was highly expressed in LUAD tissues

**Figure 1:**
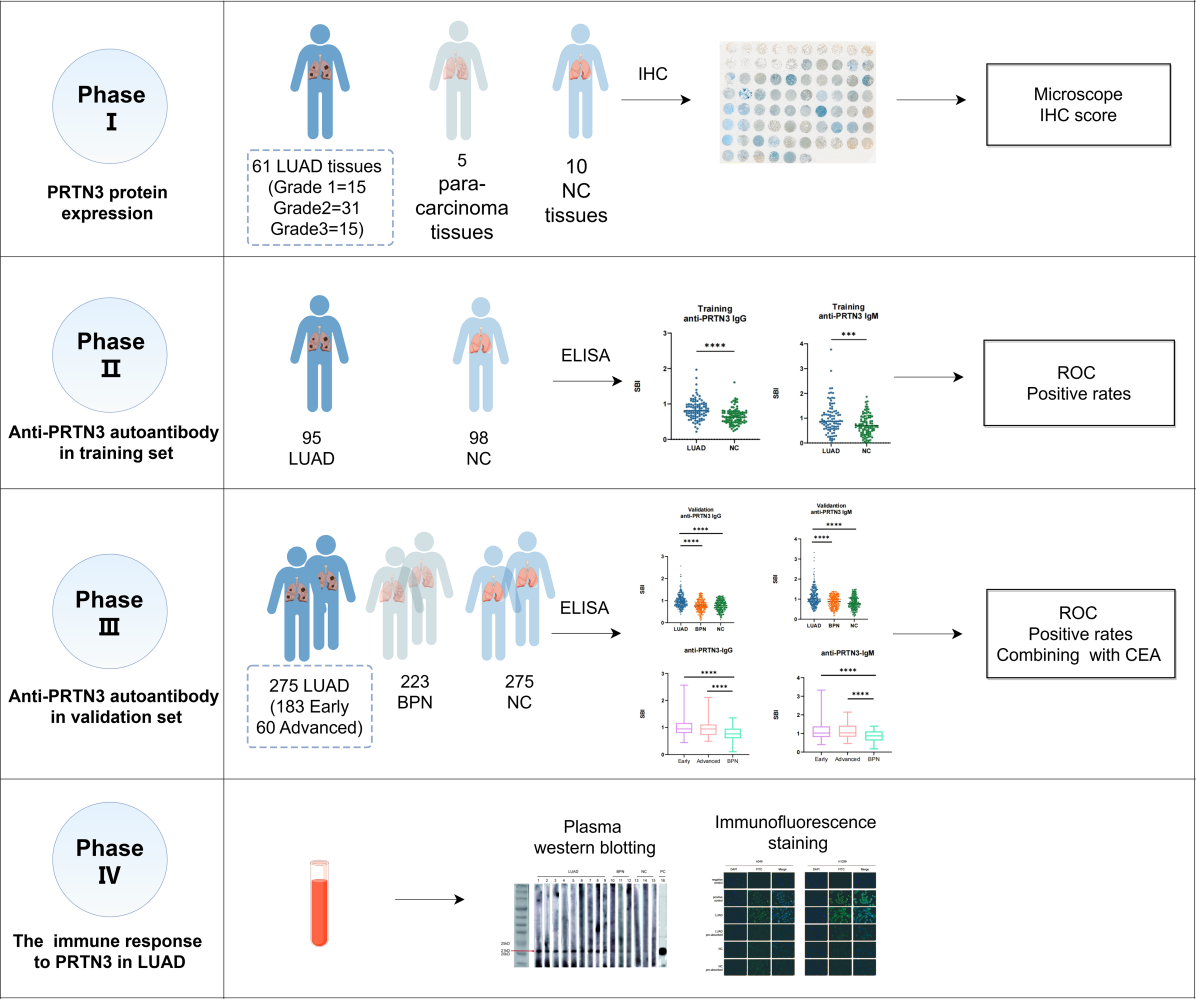
The flow diagram of this study. LUAD, lung adenocarcinoma; NC, normal control; IHC, immunohistochemistry; ELISA, enzyme-linked immunosorbent assay; BPN, benign pulmonary nodule.

**Figure 2:**
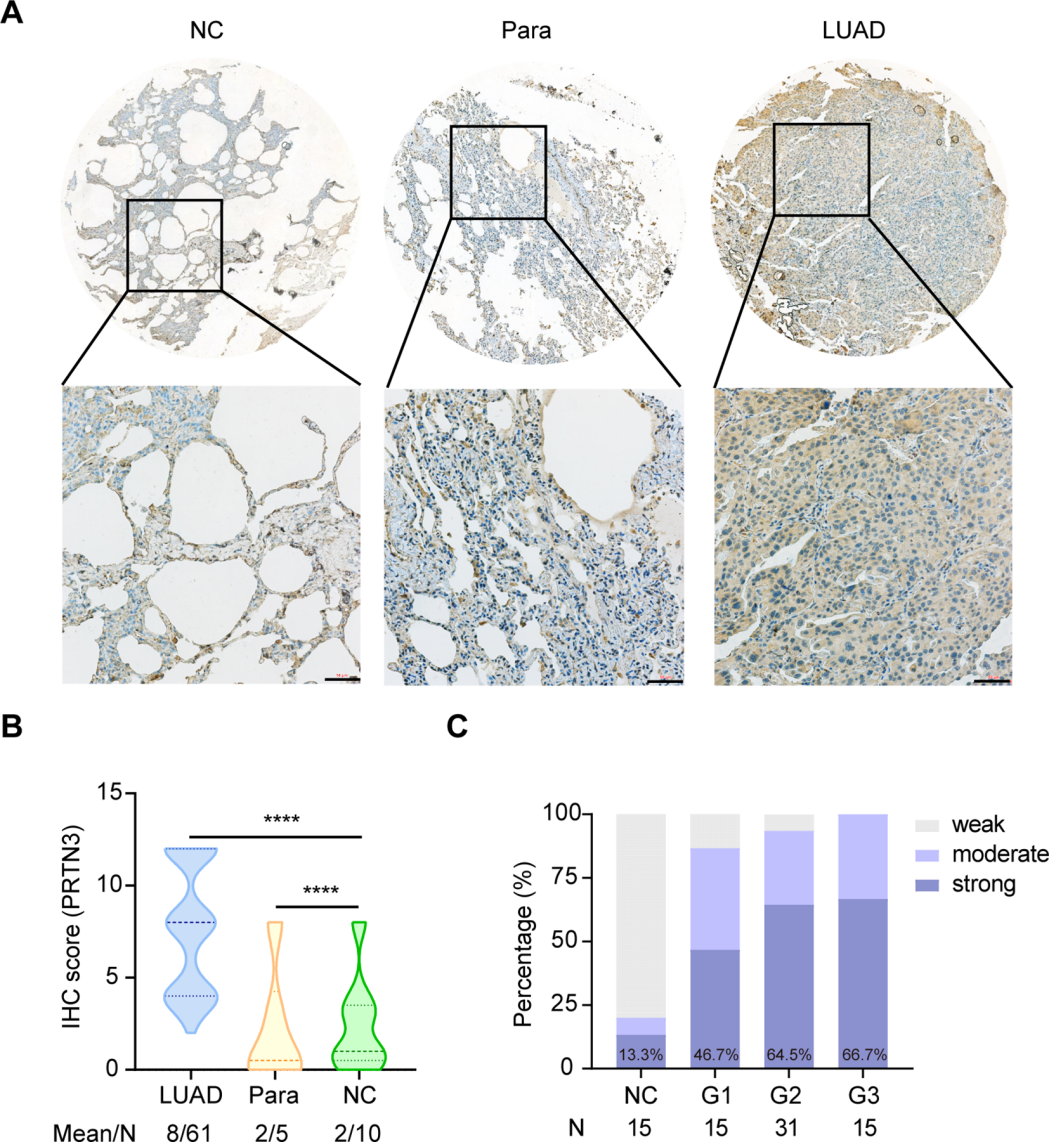
PRTN3 protein was highly expressed in LUAD tissues. **(A)** Representative IHC staining images of PRTN3 protein in NC tissue, para-cancerous tissue and LUAD tissue from tissue array (obtained at 20× by microscope). **(B)** Statistical analyses of the IHC scores of PRTN3 expression in NC tissues, para-cancerous tissues and LUAD tissues. **(C)** The expression profiles of PRTN3 in NC tissues and different pathological grades of LUAD tissues. NC, normal control; Para, para-carcinoma. ****P < 0.0001. Scale bars, 50μm. Lines represented quartile and median.

This study was conducted in four phases and aimed to investigate the autoantibodies response to PRTN3 in LUAD (**Figure 1**). We first analyzed the levels of PRTN3 protein in a tissue array containing 61 LUAD samples, 5 para-carcinoma samples, and 10 NC samples using IHC. Our findings revealed that the expression of PRTN3 protein was significantly higher in LUAD tissues compared to para-carcinoma and NC tissues (P < 0.0001) (**Figures 2A, B**). Furthermore, when considering the pathological grades (G1, G2, and G3), we observed a greater presence of strong PRTN3 expression in G2 and G3 LUAD tissues compared to G1 and NC tissues (**Figure 2C**). These results suggest that PRTN3 protein is highly expressed in LUAD tissues and positively correlates with the pathological grade, indicating its potential as an autoantigen in LUAD.

### 3.2 Plasma IgG and IgM autoantibodies against PRTN3 were significantly increased in LUAD and valuable in the diagnosis of LUAD

**Figure 3:**
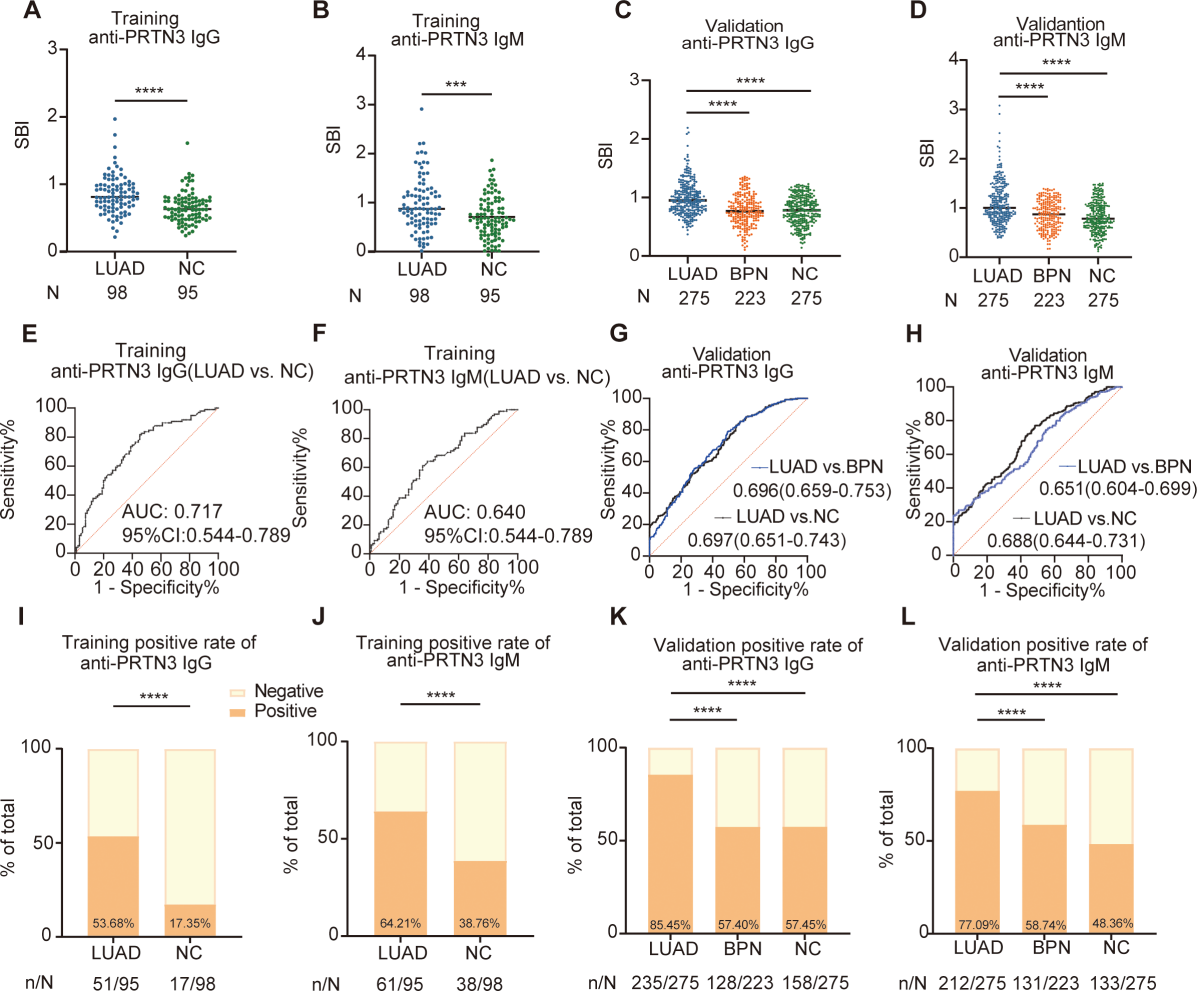
The scatter plots of the SBI and diagnostic performance of anti-PRTN3 autoantibodies in LUAD. **(A–D)** Distribution of anti-PRTN3 IgG and anti-PRTN3 IgM in the training set **(A, B)** and validation set **(C–F)** The ROC analysis of anti-PRTN3 IgG **(E)** and IgM **(F)** in differentiating LUAD from NC in the training set. **(G, H)** Diagnostic performances of anti-PRTN3 IgG **(G)** and IgM **(H)** in the validation set. **(I, J)** The positive frequency of anti-PRTN3 IgG **(I)** and IgM **(J)** in the training set. **(K, L)** The positive frequency of anti-PRTN3 IgG **(K)** and IgM **(L)** in the validation set. LUAD, lung adenocarcinoma; BPN, benign pulmonary nodule; NC, normal control; ****P < 0.0001; ***P < 0.001. Lines represented median.

We firstly examined anti-PRTN3 IgG and IgM autoantibodies in the training set, the results revealed that the expression levels of anti-PRTN3 IgG and IgM autoantibodies in LUAD were significantly higher than NC (P<0.001) (**Figures 3A, B**). To further validate these findings, we assessed the levels of autoantibodies in the validation set with a larger number of samples. We observed significantly elevated levels of IgG and IgM autoantibody responses to PRTN3 in LUAD when compared to BPN, NC (P<0.0001) (**Figures 3C, D**). These data suggest that plasma IgG and IgM autoantibodies targeting PRTN3 have the potential to serve as biomarkers for the diagnosis of LUAD.

To further evaluate their potential significance, we generated ROC curves. The ROC analysis demonstrated the ability of IgG and IgM autoantibodies targeting PRTN3 to distinguish LUAD from NC in both the training and validation sets. In the training set, the AUC for anti-PRTN3 IgG was 0.717 (95% CI: 0.644-0.789) (**Figure 3E**), and for IgM, it was 0.640 (95% CI: 0.563-0.718) (**Figure 3F**). In the validation set, with a larger sample size, the AUC of IgG in distinguishing LUAD from BPN was 0.696 (95%CI: 0.659-0.753) and from NC was 0.697 (95%CI: 0.651-0.743) (**Figure 3G**). Similarly, anti-PRTN3 IgM autoantibodies also exhibited good diagnostic efficacy in the diagnosis of LUAD, with AUC values of 0.651 (95% CI: 0.604-0.699) for distinguishing LUAD from BPN, 0.688 (95% CI: 0.644-0.731) for distinguishing from NC (**Figure 3H**). Furthermore, after defining the positive cut-off value, the distribution of positive anti-PRTN3 IgG and IgM autoantibodies was statistically analyzed. The percentage of positive anti-PRTN3 IgG (**Figures 3I, K**) and IgM (**Figures 3J, L**) autoantibodies in LUAD patients were significantly higher than in both NC and BPN groups, in both the training and validation sets (P<0.0001). Additionally, the positive predictive value (PPV) and negative predictive value (NPV) of the autoantibodies in the validation set were summarized in **Table 2**. These findings strongly indicate that anti-PRTN3 IgG and IgM autoantibodies have the potential to serve as valuable biomarkers for distinguishing LUAD from BPN and NC.

**Table 2:** Diagnostic value of anti-PRTN3 autoantibodies detected by ELISA in the validation set.

TAAb	PPV(%)	NPV(%)	PLR	NLR	FNR(%)	FPR(%)	P
NC vs. LUAD							
PRTN3-IgG	69.8	74.5	1.48	0.34	14.5	57.5	<0.0001
PRTN3-IgM	61.4	69.3	1.59	0.44	28.7	22.9	<0.0001
BPN vs. LUAD							
PRTN3-IgG	64.7	70.4	1.49	0.34	28.1	14.5	<0.0001
PRTN3-IgM	61.8	59.4	1.31	0.56	18.3	22.9	<0.0001
NC vs. BPN							
PRTN3-IgG	41.1	52.8	0.86	1.10	63.7	42.2	>0.05
PRTN3-IgM	50.4	59.9	1.25	0.82	48.9	40.7	>0.05

LUAD, lung adenocarcinoma; BPN, benign pulmonary nodule; NC, normal control; PPV, positive predictive value; NPV, negative predictive value; PLR, positive likelihood ratio; NLR, negative likelihood ratio; FNR, false negative rate; FPR, false positive rate.

These findings strongly indicate that anti-PRTN3 IgG and IgM autoantibodies have the potential to serve as valuable biomarkers for distinguishing LUAD from BPN and NC.

### 3.3 Anti-PRTN3 autoantibodies show promise as early biomarkers for LUAD

**Figure 4:**
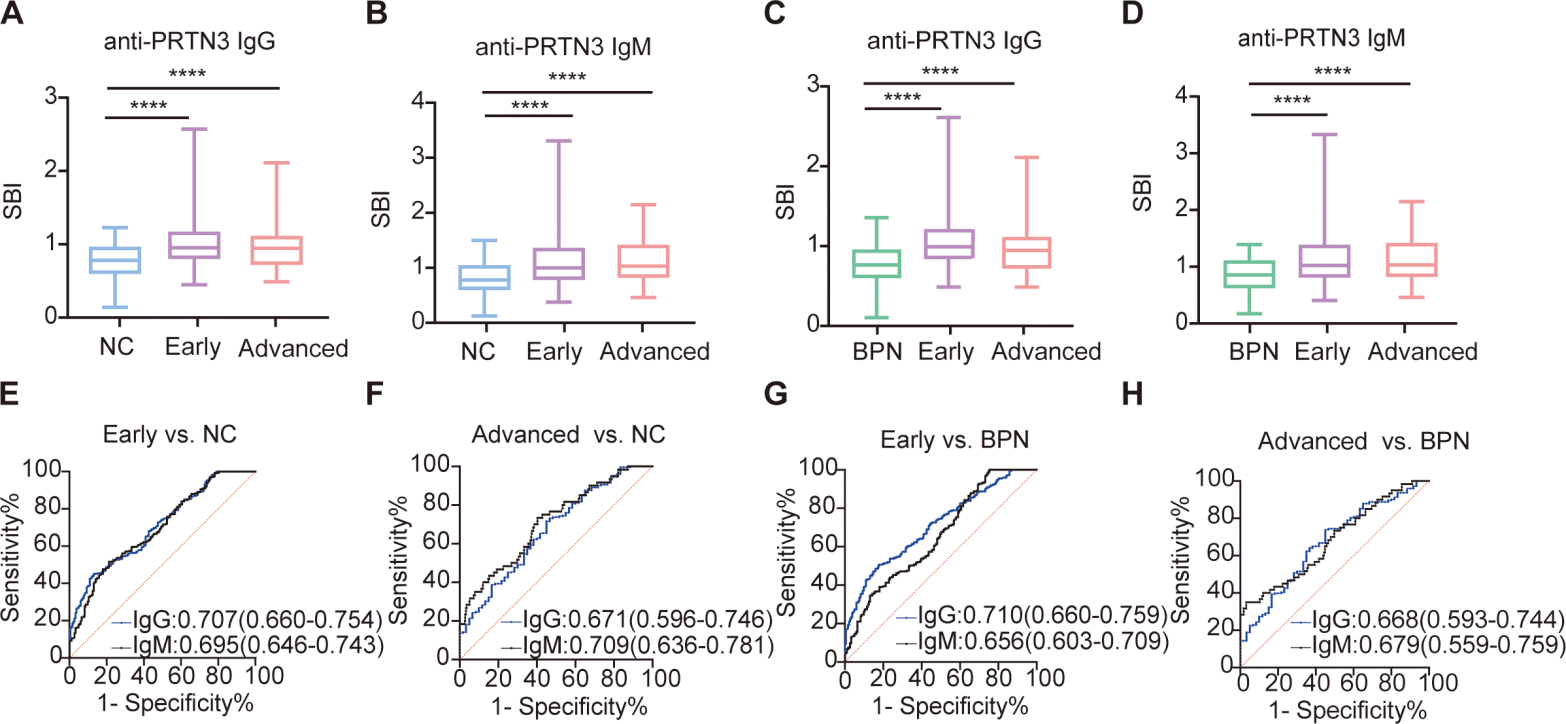
Anti-PRTN3 autoantibodies have potential diagnostic performance for early-stage LUAD. **(A-D)** Box plots present the SBI value of anti-PRTN3 IgG and IgM autoantibodies among NC **(A, B)**, BPN **(C, D)** and LUAD with early- and advanced-stage in the validation set. **(E-H)** Diagnostic performances of autoantibodies against PRTN3 for the discrimination of NC **(E, F)**, BPN **(G, H)** from LUAD with early- and advanced-stage in the validation set. AUC, area under the receiver operating characteristic curve; BPN, benign pulmonary nodule; NC, normal control; ****P < 0.0001. Lines on the boxes represent 95, 75, 50, 25, and 5 percentiles from top to bottom.

We next investigated whether anti-PRTN3 autoantibodies can serve as early diagnostic biomarkers for LUAD. Accordingly, we classified LUAD into early- stage (I+II) and advanced-stages (III+IV) based on clinical staging. The LUAD plasma samples in the validation set included 183 early-stage LUAD plasma samples and 60 advanced-stage LUAD plasma samples. Interestingly, the SBI of autoantibodies was significantly higher in early-and advanced-stage LUAD compared to both NC (**Figures 4A, B**) and BPN (**Figures 4C, D**) (P<0.0001). The AUCs for distinguishing early-stage LUAD from NC were 0.707 (95% CI: 0.660-0.754) for IgG and 0.695 (95% CI: 0.646-0.743) for IgM (**Figure 4E**). Similarly, the AUCs for distinguishing advanced-stage LUAD from NC were 0.671 (95% CI: 0.596-0.746) for IgG and 0.709 (95% CI: 0.636-0.781) for IgM (**Figure 4F**). Furthermore, the AUCs for differentiating early-stage LUAD from BPN were 0.710 (95% CI: 0.660-0.759) for IgG and 0.656 (95% CI: 0.603-0.709) for IgM (**Figure 4G**), while the AUCs for distinguishing advanced-stage LUAD from BPN were 0.668 (95% CI: 0.593-0.744) for IgG and 0.679 (95% CI: 0.559-0.759) for IgM (**Figure 4H**). Notably, there were no significant differences in SBI and AUC between early- and advanced-stage LUAD. These findings suggest that anti-PRTN3 autoantibodies could differentiate early-stage LUAD from BPN and NC.

### 3.4 Evaluation on anti-PRTN3 autoantibodies for differentiating LUAD based on clinical characteristics

We also studied the capability of anti-PRTN3 IgG and IgM autoantibodies to differentiate LUAD patients with different clinical features from BPN and NC. LUAD patients were categorized into subtypes based on TNM stage, age, sex, lymph node metastasis, distant metastasis, and tumor size. The ROC curves of anti-PRTN3 autoantibodies for diagnosing various characteristics of LUAD were depicted in **Supplementary Figures S1A–T**. The AUC values were generally similar across different subgroups, indicating that autoantibodies exhibited good diagnostic value for LUAD with different clinical characteristics (ROC>0.6). There were no statistically significant differences in the expression levels of autoantibodies between paired clinical characteristics, except for sex (**Supplementary Figures S2A, B**). These findings suggest that anti-PRTN3 autoantibodies are consistently expressed in LUAD patients with diverse clinical features.

Moreover, we investigated whether the diagnostic ability of anti-PRTN3 IgG and IgM autoantibodies for LUAD were specific to this histological subtype or if it extended to LUSC. The results revealed that the expression level of anti-PRTN3 IgG autoantibody was also significantly higher in LUSC compared to NC (P<0.0001) (**Supplementary Figure S3A**). It could differentiate LUSC from NC, the AUC for distinguishing was 0.707 (95% CI: 0.660-0.754) (**Supplementary Figure S3B**). However, anti-PRTN3 IgM autoantibody appeared to be specific only for LUAD, as it was not found to be significantly expressed at elevated levels in LUSC (**Supplementary Figure S3C**). It can distinguish LUAD from LUSC with an AUC of 0.651 (95% CI:0.680-0.772) (**Supplementary Figure S3D**). These findings indicate that while anti-PRTN3 IgG autoantibody has the potential to serve as a diagnostic biomarker not only for LUAD but also for LUSC, anti-PRTN3 IgM autoantibody can be specific to LUAD.

### 3.5 Combining anti-PRTN3 autoantibodies with CEA significantly improved the diagnostic value for LUAD

**Figure 5:**
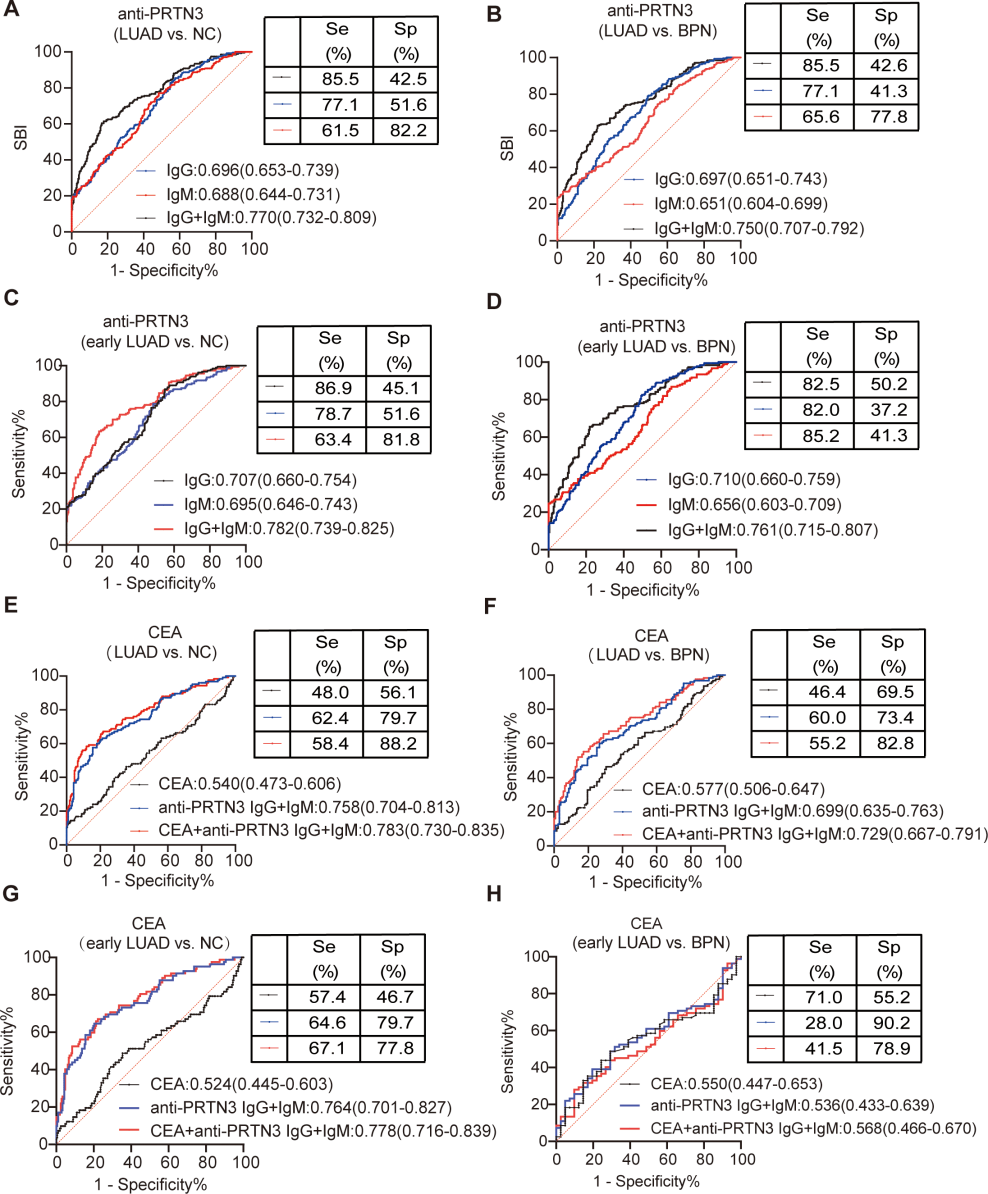
The value of combined anti-PRTN3 autoantibodies and CEA in the diagnosis of LUAD and early LUAD. **(A-D)** AUC of anti-PRTN3 IgG autoantibody, anti-PRTN3 IgM autoantibody and combination of anti-PRTN3 IgG and IgM autoantibody for the diagnosis of NC and BPN from LUAD **(A, B)** and early LUAD **(C, D)** in the validation set. **(E-H)** Diagnostic performances of CEA alone, combination of anti-PRTN3 IgG and IgM autoantibody, and the combination of anti-PRTN3 autoantibodies and CEA for the diagnosis of NC and BPN from LUAD **(E, F)** and early LUAD **(G, H)** in the validation set. LUAD, lung adenocarcinoma; BPN, benign pulmonary nodule; NC, normal control.

Currently, the combination of biomarkers and/or clinical parameters is gaining attention in research (27, 28).In our study, we compared 212 NC individuals with information on the LUAD-specific biomarker CEA, 128 BPN patients, 125 LUAD patients also with information on CEA. By utilizing binary logistic regression, we combined CEA with anti-PRTN3 IgG and IgM autoantibodies statistically. The AUC for the combined anti-PRTN3 IgG and IgM autoantibodies was 0.770 (95% CI: 0.732-0.809) for distinguishing LUAD from NC (**Figure 5A**), 0.782 (95% CI: 0.739-0.825) for distinguishing early LUAD from NC (**Figure 5C**). The AUC for the combined anti-PRTN3 IgG and IgM autoantibodies was 0.750 (95% CI: 0.707-0.792) for differentiating LUAD from BPN (**Figure 5B**), 0.761 (95% CI: 0.715-0.807) for differentiating early LUAD from BPN (**Figure 5D**). In contrast, the AUCs for CEA alone in diagnosing LUAD from NC and BPN were only 0.540 (95% CI: 0.473-0.606) and 0.577 (95% CI: 0.506-0.647), respectively (**Figures 5E, F**). When combining with anti-PRTN3 IgG and IgM autoantibodies by binary logistic regression, the AUC increases to 0.783(95% CI: 0.730-0.835) and 0.729 (95% CI: 0.667-0.791) (**Figures 5E, F**). This suggests that their combination was effective in identifying LUAD. We also found that the AUC in the diagnosis of early LUAD from NC was increased from 0.524 (95% CI: 0.445-0.603) to 0.778 (95% CI:0.716-0.839) (**Figure 5G**), and the AUC in the diagnosis of early LUAD from BPN was increased from 0.550 (95% CI: 0.447-0.653) to 0.568 (95% CI:0.466-0.670) (**Figure 5H**). These results demonstrate that anti-PRTN3 IgG and IgM autoantibodies exhibit superior diagnostic performance compared to CEA alone, showing promising potential to be biomarkers for the early diagnosis of LUAD. Their combination with CEA can also improve the diagnostic value of CEA.

### 3.6 Western blotting and IF staining confirmed the immunoreactivity of LUAD plasma to PRTN3

**Figure 6:**
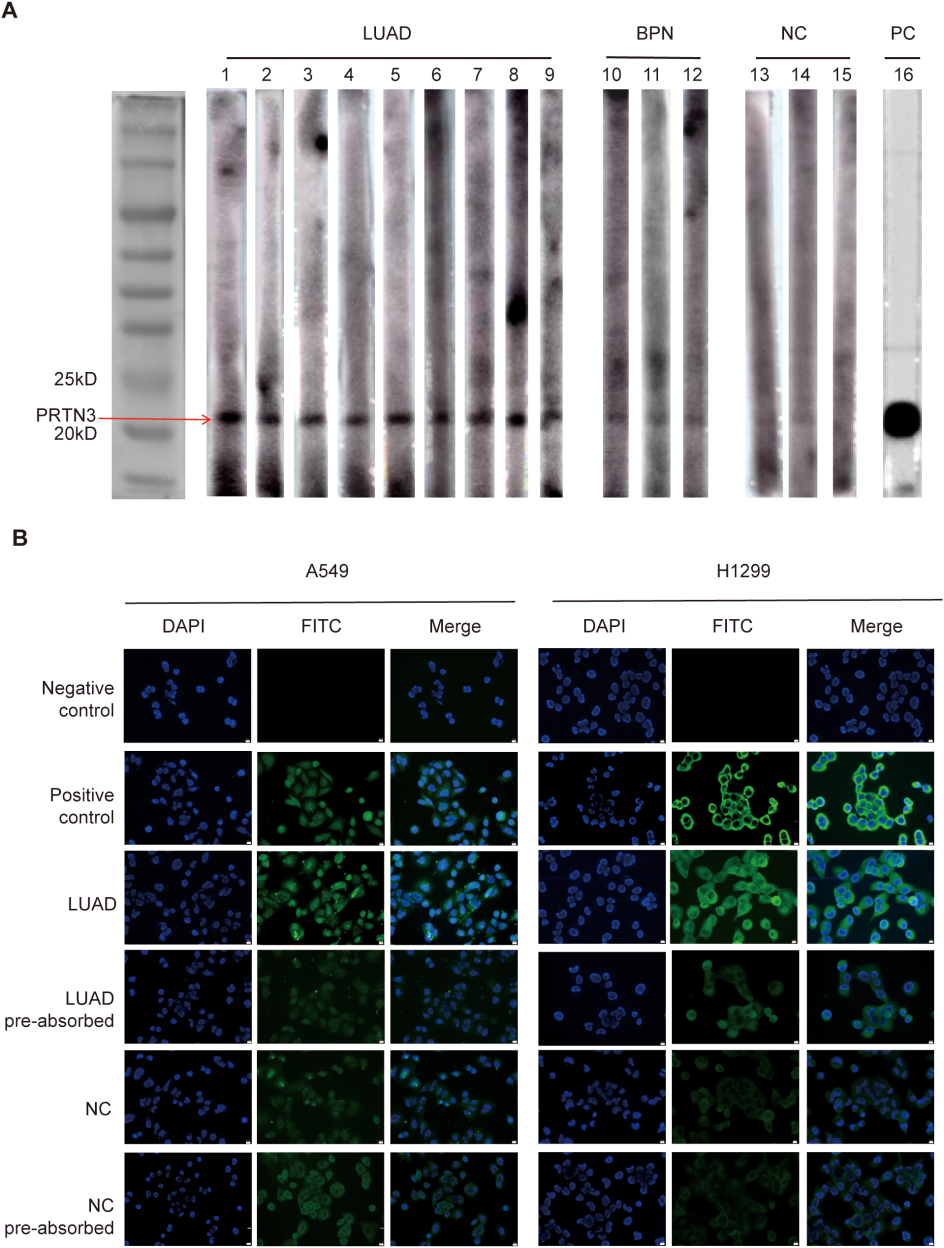
Western blotting and IF staining confirmed the immunoreactivity of LUAD plasma to PRTN3. **(A)**Western blotting of anti-PRTN3 autoantibody in human plasma. The cropping strips of lanes 1-9 representing LUAD plasma showed strong reactivity with PRTN3 recombinant protein. Lanes 10-12 (BPN) and lanes 13-15 (NC), the cropping strips of 6 random human plasma with negative reactivity to PRTN3 recombinant protein. Lane16, anti-PRTN3 antibody used as positive control. **(B)**Immunofluorescence staining of PRTN3 in LUAD cells. Phosphate-buffered saline (PBS) was used as negative control; monoclonal anti-PRTN3 autoantibody was used as positive control; a representative anti-PRTN3 autoantibody positive LUAD plasma was used as LUAD; the same LUAD plasma used in LUAD pre-absorbed was pre-absorbed with recombinant PRTN3 protein; a representative anti-PRTN3 autoantibody negative NC plasma was used as NC; the same normal control plasma used in NC pre-absorbed was pre-absorbed with recombinant PRTN3 protein, and subsequently utilized for immunofluorescence staining assay. Scale bars, 10μm. Obtained at 40× by microscope. LUAD, lung adenocarcinoma; BPN, benign pulmonary nodule; NC, normal control; PC, positive control.

To further validate the elevated levels of anti-PRTN3 autoantibodies in the plasma of LUAD patients, western blotting was performed on randomly selected plasma samples from LUAD patients, BPN patients and NC individuals. A positive control, monoclonal anti-PRTN3 antibody, was included for quality control purpose. The result, as depicted in **Figure 6A**, showed a significantly strong reaction to PRTN3 recombinant protein in all plasma samples from LUAD patients. In comparison, the plasma samples from BPN patients and NC exhibited weaker reactions with an especially low expression of anti-PRTN3 autoantibodies observed in NC plasma. The western blotting result confirmed the presence of a plasma immune response to PRTN3 and the high levels of anti-PRTN3 autoantibodies in LUAD patients’ plasma.

To examine the immunoreactivity of LUAD plasma to PRTN3 in LUAD cells, we performed IF staining. The plasma of an LUAD patient with the strongest immune response to PRTN3, as identified through western blotting, was selected as the LUAD group. Commercially available anti-PRTN3 antibodies were used for staining. In A549 cells, positive staining was observed throughout the entire cell, indicating the presence of PRTN3. In H1299 cells, cytoplasmic staining was observed, further confirming the presence of PRTN3. Furthermore, when LUAD plasma was preabsorbed by recombinant PRTN3, the IF signals in both A549 and H1299 cells were significantly reduced, indicating that the plasma of LUAD patients responded specifically to PRTN3. On the other hand, there was little change in the IF signal when NC plasma was preabsorbed by recombinant PRTN3 (**Figure 6B**). These results demonstrate the immunoreactivity of LUAD plasma to PRTN3 in LUAD cells.

## 4 Discussion

Recent studies have suggested that plasma TAAbs may hold promise as cost-effective early biomarkers for cancer diagnosis and distinguishing between benign and malignant pulmonary nodules (29–31). Among these TAAbs, PRTN3 is of particular interest. It is stored in its active form within neutrophil azurophilic granules and is involved in regulating inflammation (32). Notably, PRTN3 has been found to be overexpressed in early-stage cancers. Despite this, there is limited research on the role of anti-PRTN3 autoantibodies in tumors. To our knowledge, our study is the first to investigate the early diagnostic value of anti-PRTN3 IgG and IgM autoantibodies in LUAD.

Our study revealed elevated levels of PRTN3 protein in LUAD tissues, indicating its involvement in the development of LUAD and its potential as a TAA specific to LUAD. TAAs are known to be secreted into the bloodstream, inducing an immune response and leading to the production of detectable TAAbs. In line with this, our ELISA results demonstrated that anti-PRTN3 IgG and IgM autoantibodies were highly expressed in LUAD patients, highlighting their diagnostic potential for LUAD and their ability to differentiate between benign and malignant pulmonary nodules. It is worth noting that the diagnostic value of IgM antibody was slightly lower than that of IgG antibody. This could be attributed to the fact that IgM production is typically reduced during the development of an IgG response and is generally considered to play a less prominent role in long-term immunity (33, 34).

Furthermore, we observed significantly increased expression levels of anti-PRTN3 IgG and IgM autoantibodies in the early-stage of LUAD, which aligns with the characteristic appearance of autoantibodies during the early-stages of cancer (10). This suggests that these autoantibodies may serve as early biomarkers for LUAD. Next, our findings support the notion that anti-PRTN3 IgG and IgM autoantibodies have the potential to serve as valuable biomarkers for the early detection and diagnosis of LUAD. The AUC of anti-PRTN3 autoantibody in the diagnosis of early LUAD from NC was 0.782 (95% CI: 0.739-0.825), and the sensitivity and specificity were 59.4% and 88.2%, respectively. Again, the AUC of early LUAD from BPN was 0.761 (95% CI: 0.715-0.807) and the sensitivity and specificity were 85.2% and 43.1%.

In recent years, there has been growing interest in combining TMs and autoantibodies to improve diagnostic efficiency in cancer diagnosis (35–38). In our study, we found that the model combining anti-PRTN3 IgG, IgM, and CEA improved diagnostic value of CEA alone for LUAD and early LUAD. In LUAD diagnosis, this model yielded an AUC of 0.783 (95% CI: 0.730-0.835) for NC diagnosis, with a sensitivity of 58.4% and a specificity of 88.2%. Additionally, the diagnostic efficiency for BPN was significantly improved, with an AUC of 0.729 (95% CI: 0.667-0.791), a sensitivity of 55.2%, and a specificity of 82.8%. For the early diagnosis of LUAD, the AUC for NC was 0.778 (95% CI: 0.716-0.839), sensitivity was 67.1%, specificity was 77.8%. The AUC for BPN was 0.568 (95% CI: 0.466-0.670). Its AUC lower than 0.761 of anti-PRTN3 autoantibodies diagnosing early LUAD from BPN may be due to the small number of patients with early LUAD who had CEA information. These findings have the potential to contribute to the development of new diagnostic strategies for LUAD that can improve patient outcomes. This approach is characterized by its simplicity, efficiency, and minimally invasive nature.

The use of TAAbs as early diagnostic biomarkers poses challenges due to the variability in immunogenicity among patients with different clinical characteristics (39). However, in our study, we found that only women had significantly higher levels of anti-PRTN3 autoantibodies compared to men (P<0.0001). This observation may be attributed to the fact that women generally exhibit stronger immune responses than men, and estrogen receptor expression can enhance both cellular and humoral immunity. Conversely, androgen has been shown to inhibit immune cell activity (40). Interestingly, no significant differences were observed in the expression levels of anti-PRTN3 IgG and IgM autoantibodies between other pairs of clinical characteristics, such as early-stage versus advanced-stage disease. This suggests that anti-PRTN3 autoantibodies are relatively stable and reliable compared to traditional TM, thereby offering broader application prospects. Overall, our findings indicate that anti-PRTN3 autoantibodies exhibit stability and reliability across different clinical characteristics, with the exception of higher levels observed in women. This highlights their potential as robust biomarkers for the diagnosis of LUAD.

Our study does have certain limitations that should be acknowledged. Firstly, the sampling method employed in our study was biased, as we used plasma samples solely from a single hospital and did not establish a population-based cohort. As such, further investigations involving population-based multicenter studies are warranted to validate the potential of plasma anti-PRTN3 autoantibodies as biomarkers for LUAD. Secondly, due to the focus on diagnostic value, we lacked a prognostic analysis of the LUAD patients included in our study. It has been suggested that autoantibodies may play a protective role in certain cases (41), and their presence has been associated with better prognosis (42, 43). Therefore, future studies should consider exploring the prognostic implications of anti-PRTN3 autoantibodies in LUAD patients, which could provide valuable insights into the clinical significance and potential therapeutic implications of these autoantibodies.

## 5 Conclusion

In summary, our study demonstrates that the levels of both anti-PRTN3 IgG and IgM autoantibodies in the plasma of LUAD patients are significantly higher compared to those in BPN and NC. This finding align with the elevated expression of the corresponding PRTN3 antigen observed in LUAD tissues. Notably, anti-PRTN3 IgG and IgM autoantibodies exhibit the ability to differentiate LUAD patients with early-stage from those with BPN and NC. Overall, our study highlights the clinical utility of anti-PRTN3 IgG and IgM autoantibodies as potential early diagnostic markers for LUAD to complement current diagnostic methods.

## Data Availability

The raw data supporting the conclusions of this article will be made available by the authors, without undue reservation.
