# Supplementary material for: Plasma anti-PRTN3 IgG and IgM autoantibodies: novel biomarkers for early detection of lung adenocarcinoma
Source: Front Immunol. 2025 Feb 14;16:1534078. doi: 10.3389/fimmu.2025.1534078 (PMC11868074; doi:10.3389/fimmu.2025.1534078)
Supplement: Supplementary file 1 [file DataSheet1.docx]

Supplementary Material

## Supplementary Figure

##
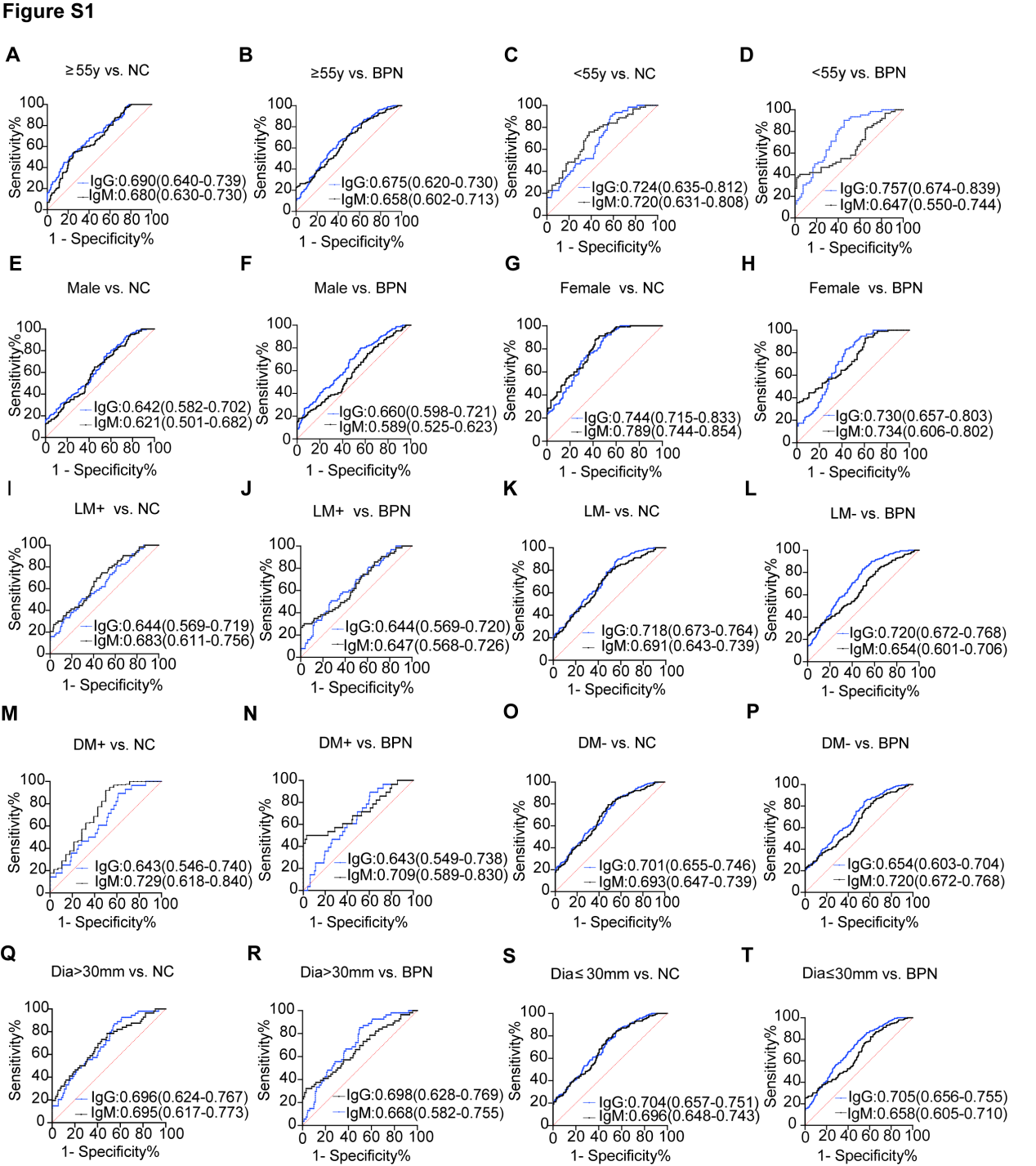


## **Supplementary Figure 1.** Diagnostic performances of anti-PRTN3 autoantibodies in different clinical characteristics of LUAD. The clinical characteristics included age≥55y **(A, B)** and <55y **(C, D)**, male **(E, F)** and female **(G, H)**, lymphatic metastasis (+) **(I, J)** and lymphatic metastasis (-) **(K, L)**, distance metastasis (+) **(M, N)** and distance metastasis (-) **(O, P)**, tumor diameter＞30mm **(Q, R)** and tumor diameter ≤ 30mm **(S,T)**. BPN, benign pulmonary nodule; NC, normal control.


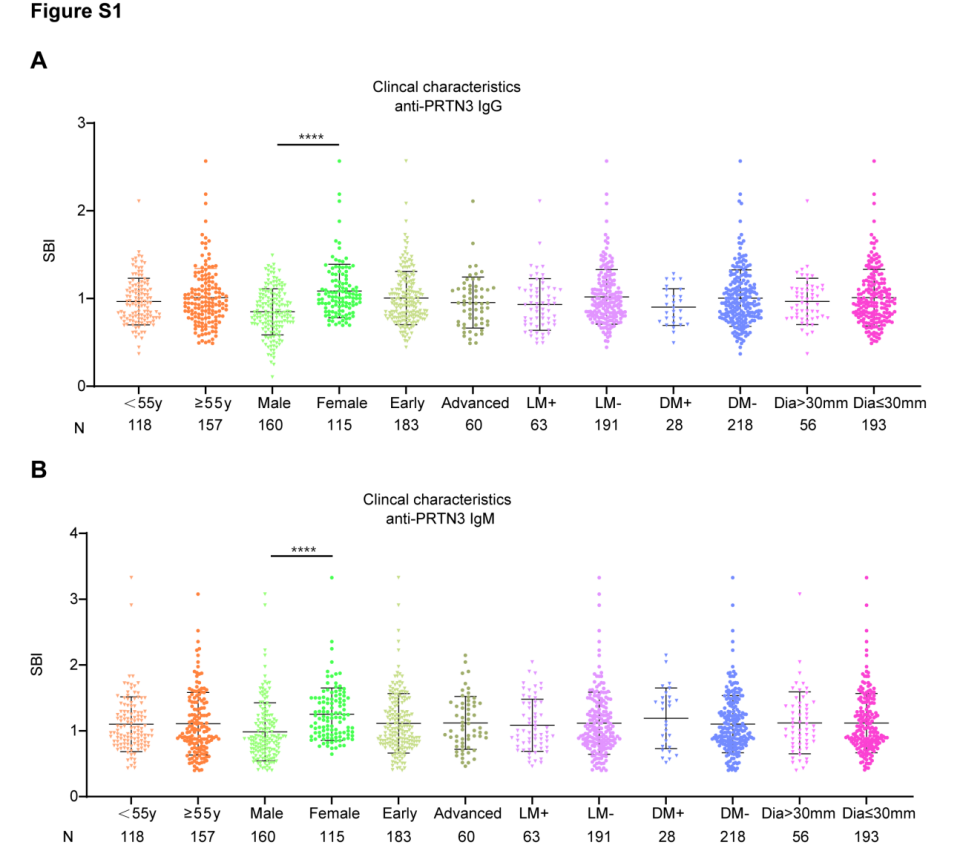


**Figure S2.** The SBI of anti-PRTN3 autoantibodies between pairwise clinical characteristics. **(A, B)** Violin plots show the expression levels of anti-PRTN3 IgG **(A)** autoantibody and anti-PRTN3 IgM **(B)** autoantibody in LUAD patients with pairwise clinical features. LM+, lymphatic metastasis (+); LM-, lymphatic metastasis (-); DM+, distance metastasis (+); DM-, distance metastasis (-); Dia, tumor diameter. ****P < 0.0001. Lines represented mean ± SD.


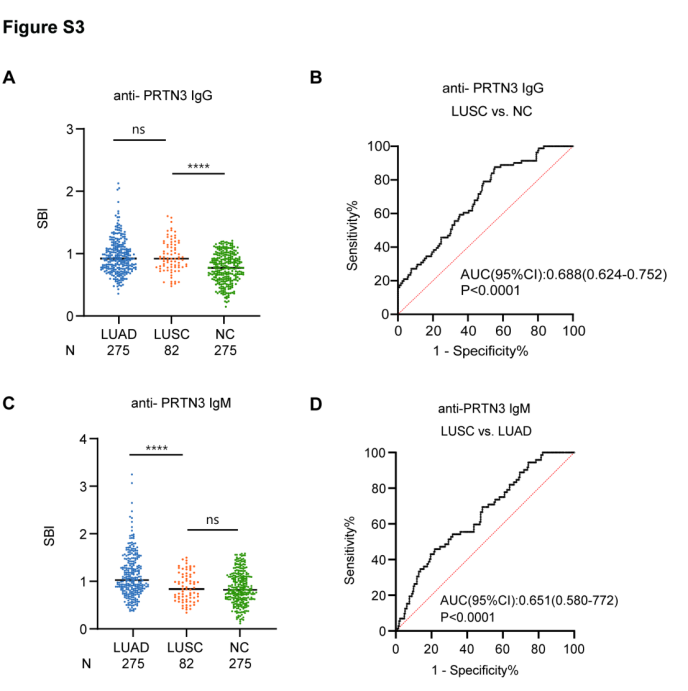


**Figure S3.** Anti-PRTN3 IgM can specifically diagnose LUAD and distinguish it from LUSC. **(A, C)** The distribution of plasma anti-PRTN3 IgG **(A)** and anti-PRTN3 IgM **(C)** in LUAD, LUSC and NC in the validation set. **(B)** Diagnostic performances of anti-PRTN3 IgG in differentiating NC from LUSC in the validation set. **(D)** Diagnostic performances of anti-PRTN3 IgM in differentiating LUAD from LUSC in the validation set. AUC, area under the receiver operating characteristic curve; LUAD, lung adenocarcinoma; LUSC, lung squamous cell carcinoma; NC, normal control; ****P < 0.0001; ns, no significance. Lines represented median.
