# Supplementary figures and images for: Plasma anti-PRTN3 IgG and IgM autoantibodies: novel biomarkers for early detection of lung adenocarcinoma
Source: Front Immunol. 2025 Feb 14;16:1534078. doi: 10.3389/fimmu.2025.1534078 (PMC11868074; doi:10.3389/fimmu.2025.1534078)

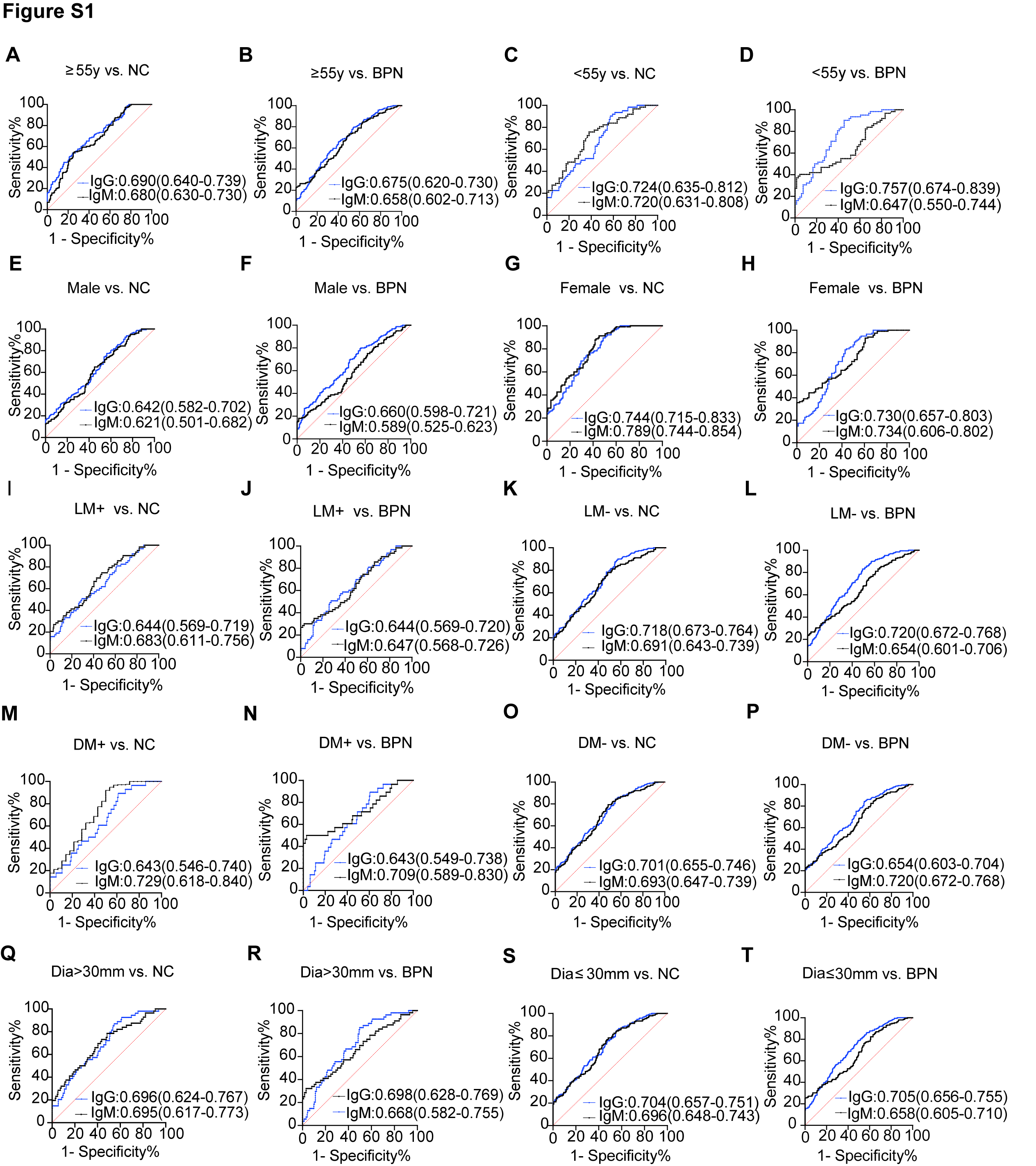

Supplement: Supplementary file 2 [file Image1.tif]

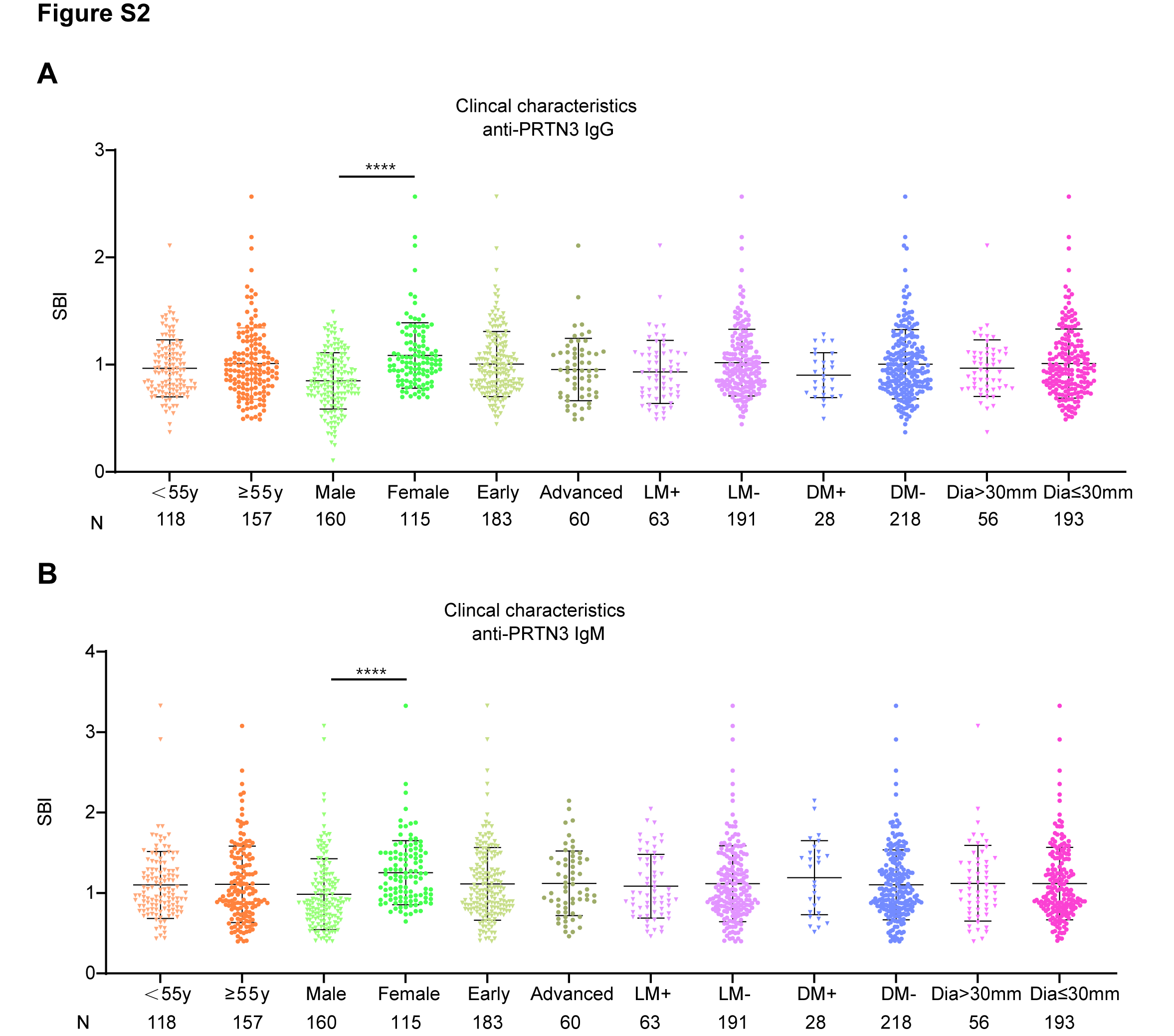

Supplement: Supplementary file 3 [file Image2.tif]

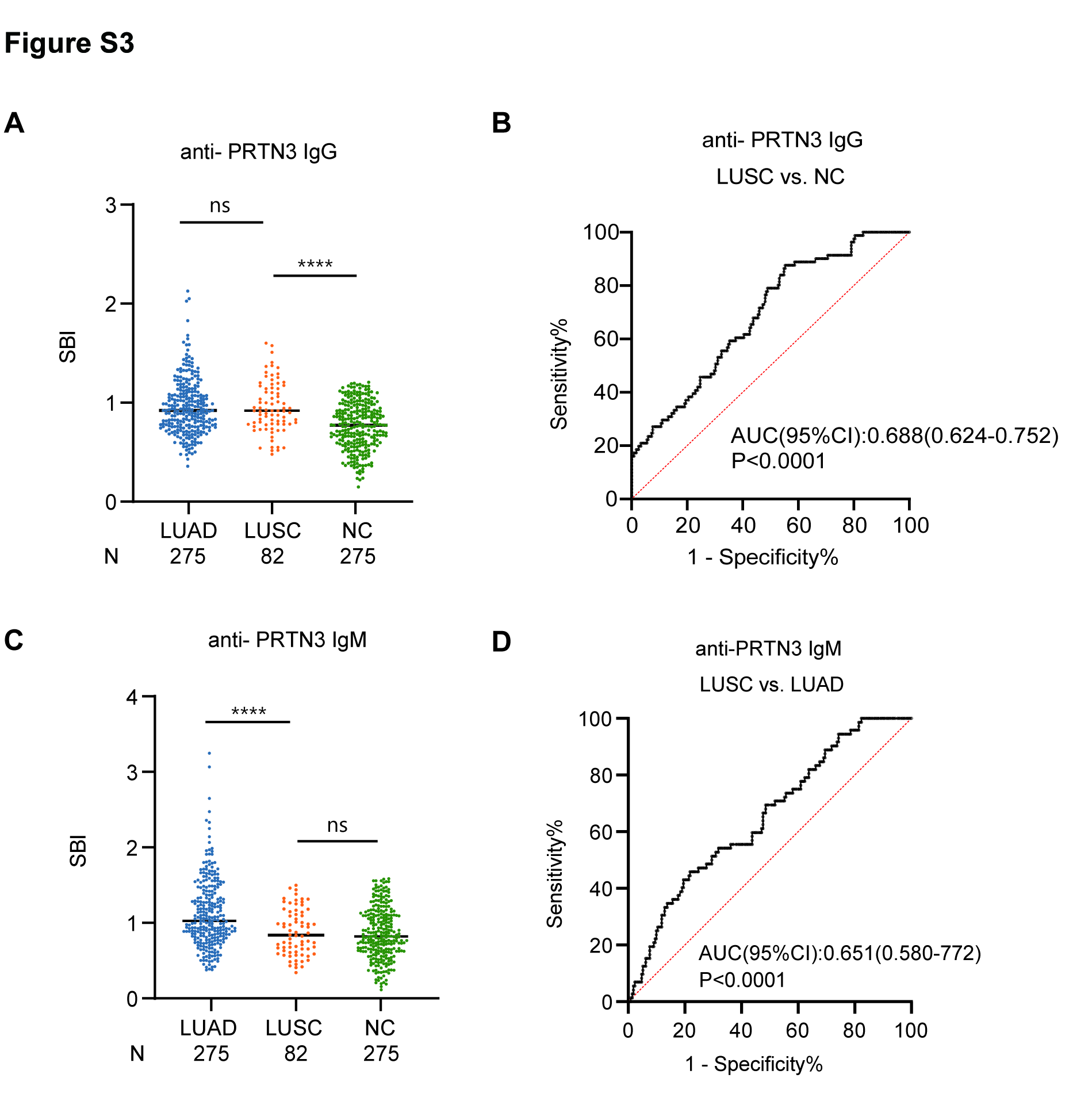

Supplement: Supplementary file 4 [file Image3.tif]
